# The Influence of Low Energy Availability on Bone Mineral Density and Trabecular Bone Microarchitecture of Pubescent Female Athletes: A Preliminary Study

**DOI:** 10.3390/ijerph19095580

**Published:** 2022-05-04

**Authors:** Nodoka Ikegami, Mina Samukawa, Mikako Sakamaki-Sunaga, Makoto Sugawara, Shizuka Torashima, Tomoya Ishida, Satoshi Kasahara, Harukazu Tohyama

**Affiliations:** 1 Department of Exercise Physiology, Nippon Sport Science University, Tokyo 158-8508, Japan; ikegami-n@nittai.ac.jp (N.I.); sunaga@nittai.ac.jp (M.S.-S.); 2 Faculty of Health Sciences, Hokkaido University, Sapporo 060-0812, Japan; t.ishida@hs.hokudai.ac.jp (T.I.); kasahara@hs.hokudai.ac.jp (S.K.); tohyama@med.hokudai.ac.jp (H.T.); 3 Matsuda Orthopedic Memorial Hospital, Sapporo 001-0018, Japan; sugawara@matsuda-oh.com; 4 Iwamizawa Campus Arts and Sports Couse, Hokkaido University of Education, Iwamizawa 068-0835, Japan; shizukatorashima@gmail.com

**Keywords:** female athlete triad syndrome, relative energy deficiency, adolescent, ideal body weight, cancellous bone

## Abstract

The influence of low energy availability (LEA) on bone mineral density (BMD) and trabecular bone microarchitecture in pubescent female athletes is unclear. This study aimed to investigate the influence of LEA on BMD and trabecular bone microarchitecture in 21 pubescent female athletes (age, 12–15 years; 11 track and field athletes, 10 gymnasts). We used two indices to assess LEA: energy availability and the percent of ideal body weight. Dual-energy X-ray absorptiometry was used to obtain total body less head, lumbar spine BMD Z-scores, and lumbar trabecular bone scores (TBS). Pearson’s or Spearman’s correlation coefficients were used to assess the relationship among EA, percent of ideal body weight, and bone parameters. The threshold for statistical significance was set at *p* < 0.05. The percent of ideal body weight was significantly correlated with the BMD Z-scores of the total body less head (r = 0.61; *p* < 0.01), lumbar spine (r = 0.55; *p* < 0.01), and lumbar TBS (r = 0.47; *p* = 0.03). However, energy availability was not correlated with bone parameters. These findings suggest that screening for low ideal body weight may be a useful predictor of low BMD and insufficient trabecular bone microarchitecture in pubescent female athletes.

## 1. Introduction

Low energy availability (LEA) is defined as a deficiency in energy intake compared with energy expenditure during exercise. High prevalence rates of LEA (31 to 63%) have been reported in female athletes [1,2,3]. The American College of Sports Medicine (ACSM) suggested that the female athlete triad (FAT) refers to the three most common health problems in female athletes: LEA, menstrual dysfunction, and low bone mineral density (BMD) [4,5]. The International Olympic Committee has also stated that relative energy deficiency in sport could lead to FAT-related health issues and negatively affect athletic performance [6]. Taken together, appropriate energy status is important for female athletes’ health and performance.

Low energy availability has been associated with the suppression of metabolic and reproductive hormones that cause bone impairment [7,8,9]. A previous study revealed that 5-day protocols of restricted energy availability (EA) decreased bone formation and increased bone resorption in females [9]. The ACSM defines LEA screening as a body mass index of less than 18.5 kg/m^2^ in adult athletes, and an actual body weight of less than 90% of the ideal body weight in adolescent athletes [4]. Several studies have demonstrated that low percent of ideal body weight and BMI leads to low BMD and stress fractures in female athletes [10,11,12,13]. However, the age of the participants in these previous studies was higher than that of high school athletes.

Bone mass accrual peaks between 11–14 years of age and then sharply declines after 16–18 years of age, as shown by longitudinal and cross-sectional studies [14,15]. Thus, the influence of LEA on bone health in pubescent female athletes needs to be clarified. Furthermore, 16–17 years of age is reported to be the most susceptible time for stress fractures [16]. As such, preventing low BMD among pubescent athletes should be considered vital; however, studies on the influence of LEA on bone health in pubescent female athletes are limited. Therefore, such data may provide evidence to support the prevention of low BMD and stress fractures in female athletes.

Although the measurement of BMD with dual-energy X-ray absorptiometry (DXA) has been mainly used for the assessment of osteoporosis, it has recently been thought to be insufficient to assess bone health using this two-dimensional measurement only. Previous studies have revealed that the evaluation of the microarchitecture of bone structure might significantly enhance the accuracy of bone strength and fracture risk in older populations [17,18]. In addition, adolescents with anorexia nervosa had lower trabecular bone density than controls, although their BMD did not differ [19,20]. Thus, the assessment of trabecular bone microarchitecture can provide detailed information on bone health, and there is no research examining trabecular bone microarchitecture in pubescent female athletes.

This study aimed to investigate the effect of LEA on BMD and trabecular bone microarchitecture in pubescent female athletes. We hypothesized that LEA negatively affects BMD and trabecular bone microarchitecture in pubescent female athletes.

## 2. Materials and Methods

### 2.1. Participants

Twenty-one pubescent female athletes aged 12–15 years participated in the study. Eleven participants were track and field athletes and 10 were gymnasts. Individuals who regularly practiced sports for >150 min per week were recruited through local sports club teams. The number of years of current sports experience of participants ranged from 4.5 to 11.0 years. We excluded participants taking medication that could affect BMD. This research was approved by the institutional review board of the authors’ affiliated institution (approval code: 19–39). All athletes and their guardians signed informed consent forms in accordance with the Declaration of Helsinki before participating in this study. This study was conducted between October 2019 and March 2020.

### 2.2. Study Design

This was a cross-sectional study. Body components, maturity status, EA, total body less head (TBLH) and lumbar BMD (with DXA), and lumbar trabecular bone microarchitecture of each participant were assessed. In addition, all participants completed a questionnaire about sports participation, menstrual status, history of stress fractures, medication intake, diet, and disordered eating (EAT-26).

### 2.3. Evaluation of Maturity Status

The height, body weight, and sitting height of the participants were measured. The height was measured with a standard stadiometer to the nearest 0.1 cm. The sitting height was also measured by the stadiometer on a standardized plinth. The body mass was measured by a standard weight scale to the nearest 0.1 kg. These anthropometric data were used to calculate the maturity offset, which estimates the years from peak height velocity. We used the formula proposed by Mirwald et al. [21].

### 2.4. Evaluation of LEA

Low energy availability was defined as less than 30 kcal/kg fat-free mass (FFM) and less than 90% of ideal body weight by the ACSM statement [4], and the same criteria were used in this study. Energy intake was calculated by recording daily food intake over a 3-day period [22]. Participants were asked to keep records of all food and beverages consumed for three days (two training days and one resting day). The data were analysed using nutrition analysis software (Nutrition Advice Personal Computer System, Central Officemation Corporation, Sapporo, Japan), and the energy intake was measured in kilocalories. In addition to their dietary records, participants were also asked to keep training logs [1] in which they recorded the types of exercises and their training time. Energy expenditure was analysed by calculating metabolic equivalents [23], and the EA was then calculated using the formula below [4]. We used FFM data from the DXA measurements.

EA = (mean daily energy intake (kcal) − mean daily exercise energy expenditure (kcal))/FFM (kg)

The ideal body weight was calculated using a formula obtained from the Japanese Society for Pediatric Endocrinology (http://jspe.umin.jp/medical/taikaku.html, accessed on 4 May 2022).

### 2.5. Evaluation of BMD and Trabecular Bone Microarchitecture

The BMD and body components were measured using DXA (Horizon-Wi, Hologic. Marlborough, MA, USA). Images were taken of the lumbar spine (L2–L4) and TBLH. The BMD Z-scores were used with their ages matched, together with sex-specific reference data from the Hologic Pediatric Database [24]. For the lumbar spine, we referred to the Japanese Hologic Pediatric Database. The Z-scores < −1.0 were used for the diagnosis of low BMD in female athletes involved in regular weight-bearing sports [4,5,25].

The trabecular bone score (TBS) is obtained from the lumbar spines of DXA images using variations in grayscale and texture, which in turn can provide a quantitative estimate of trabecular microarchitecture [26]. The TBS of the lumbar vertebrae was determined using the TBS Insight Software (Medimaps, Switzerland).

### 2.6. Statistical Analysis

The software SPSS Statistics version 27.0.0.1 (IBM Corp., Armonk, NY, USA) was used for all statistical analyses. The Shapiro–Wilk test was performed to determine the normality of data distribution. Pearson’s correlations of the percent of ideal body weight and EA with BMD Z-scores and TBS were determined. Potential differences between the LEA groups were determined using the independent *t*-test or Mann–Whitney U test. The relationship between energy-related parameters (EAT-26, energy intake, exercise-induced energy expenditure), physical parameters (body weight, FFM, fat mass, and maturity offset), and bone parameters was evaluated using Pearson’s correlation or Spearman’s correlation coefficients. All data were used for correlation analysis. The significance level was set at *p* < 0.05.

## 3. Results

The participant characteristics are shown in Table 1. Six participants (28.6%, three athletes and three gymnasts) had less than 90% ideal body weight, and twelve (57.1%, eight athletes and four gymnasts) had less than 30 kcal/kg FFM. Twelve participants (57.1%) had experienced the onset of menarche. None of the patients had current secondary amenorrhea, although three had a history of secondary amenorrhea. Only one participant met the criteria for low BMD (Z-score < −1), and four had a history of stress fractures in the tibia (*n* = 2) and lumbar spine (*n* = 2). Fourteen participants (66.7%) worried about their weight, and four (19.0%) were on a restrictive diet.

**Figure 1:**
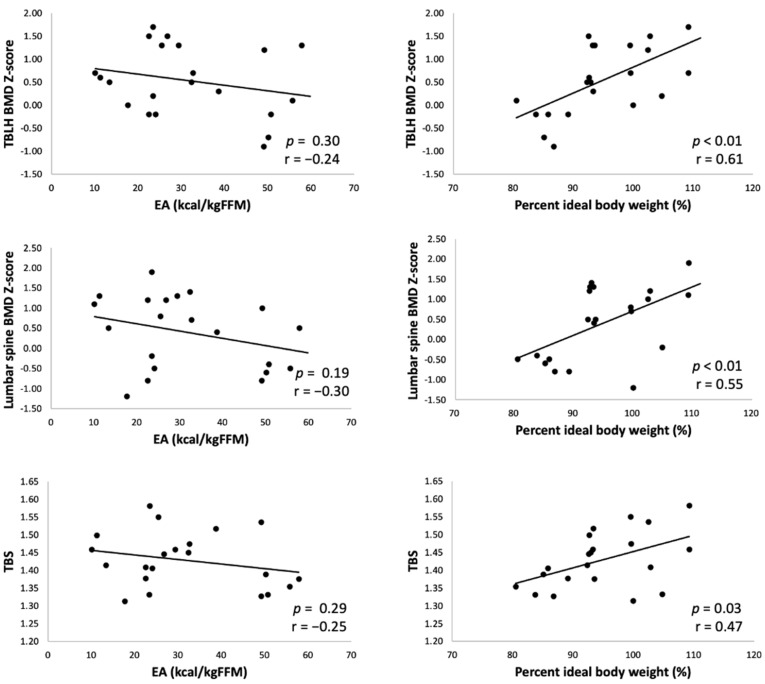
Correlations among bone parameters, EA, and percent of ideal body weight. Percent of ideal body weight was significantly correlated with BMD Z-scores of the TBLH (r = 0.61; *p* < 0.01), lumbar spine (r = 0.55; *p* < 0.01), and lumbar TBS (r = 0.47; *p* = 0.03).

Figure 1 shows the association between LEA and bone parameters. The percent of ideal body weight was significantly correlated with the BMD Z-scores of the TBLH (r = 0.61; *p* < 0.01), lumbar spine (r = 0.55; *p* < 0.01), and lumbar TBS (r = 0.47; *p* = 0.03). However, no correlations were found between EA and the bone parameters. Table 2 shows the characteristics of athletes according to each LEA definition: body weight <90% of the ideal body weight and EA <30 kcal/FFM, respectively. Table 3 shows the correlations between bone parameters and physical and energy characteristics. Other parameters related to LEA (energy intake, exercise energy expenditure, and EAT-26) were not correlated with any bone parameters. The FFM mass was significantly correlated with all bone parameters (*p* < 0.05). Body weight was correlated with BMD Z-scores but not with TBS. 

## 4. Discussion

We investigated the relationship between LEA, BMD, and trabecular bone microarchitecture in pubescent female athletes. The percent of ideal body weight was negatively associated with both BMD and TBS; however, EA was not significantly associated with bone parameters. These findings partly support our hypothesis that LEA negatively affects bone health in pubescent female athletes.

Low body weight (<90% ideal body weight) and low BMI (<18.5 kg/m^2^) were reported as risk factors for low BMD among exercising females [11] and high-school distance runners [13,27]. The present results showed that LEA negatively affected the BMD in pubescent female athletes. Nose-Ogura et al. [12] found that teenage female athletes with low body weight (<85% ideal body weight) were at significant risk of experiencing types of stress fractures not seen in female athletes over 20 years old. Loucks et al. [28] also reported that LEA significantly reduced luteinizing hormone and growth hormone levels in adolescents compared to those in adults. As puberty is a particularly critical time for teenagers regarding bone mineral accrual [14,15], pubescent female athletes are potentially at risk of LEA with regard to their BMD.

In addition, we demonstrated that the percent of ideal body weight can negatively affect the lumbar TBS in pubescent female athletes. The trabecular bone score reflects the trabecular bone microarchitecture [29,30]. Measurements of BMD are mainly influenced by cortical bones, which have a lower bone remodelling process than trabecular bones [31]. In the present study, no correlation was observed between the TBS and body weight. The FAT risk factors in female collegiate athletes were significantly associated with lower TBS values [32]. The TBS was also affected by the effects of training loading as well as BMD. However, a previous study reported that the TBS values of endurance runners were the lowest among various sports types, and the scores were significantly lower than that of swimmers or non-athletes, even though their lumbar BMD did not differ between endurance runners and non-athletes [33]. Thus, the TBS may be more susceptible to LEA than BMD. However, this is only the first attempt to assess TBS values in pubescent female athletes, and further examination is required.

In this study, energy parameters were not significantly correlated with bone parameters. Furthermore, none of the nutritional variables, including calcium, from the 3-day food diary were associated with bone parameters. Previous studies have also reported that there were no significant differences in energy intake, energy expenditure, and EA between amenorrheic athletes and eumenorrheic athletes, even though the BMD is lower in amenorrheic athletes than in eumenorrheic athletes [1,22]. Metabolic and reproductive hormones are suppressed and bone turnover is immediately elevated when participants are controlled to reduce EA in the laboratory [7,8]. However, it takes considerably longer for the effects of LEA on BMD to be revealed [4,34], and as such, we could not find any effect of EA in a short-term assessment of BMD. Among the participants of this study, 66.7% were concerned about their weight and 57.1% were taking less than 30 kcal/kg FFM. Additionally, the group with EA < 30 kcal/kg FFM was significantly heavier and had a greater percentage of body fat than the group with EA ≥ 30 kcal/kg FFM. Such concerns about body weight affected the self-reported EA assessment in this study. Therefore, a more objective assessment is needed to assess LEA and predict low BMD in paediatric athletes.

To the best of our knowledge, this is the first study to investigate the relationship between LEA, BMD, and TBS in pubescent female athletes. Our findings suggest that LEA indicated by the percent of ideal body weight can affect BMD and TBS. Adolescence is a critical time for bone mineral acquisition [14,15], and “catch-up” accrual is difficult thereafter [35]. Maximizing bone mass during growth is important not only for preventing stress fractures but also for preserving bone health throughout one’s lifespan. Even though high-impact exercises have been shown to confer positive effects on bone health, continuous LEA status during puberty may lead to poor bone health [36,37,38]. Furthermore, in adult females, menstrual dysfunction is considered a useful predictor of low BMD [11,13,27,39]. However, menstrual dysfunction commonly occurs within several years after menarche; therefore, predicting low BMD from the menstrual status of pubescent females may be difficult. Further investigation is required to develop an effective screening technique for the prevention of low BMD and impaired bone microarchitecture in female pubescent athletes.

In this study, female pubescent athletes had low body weight and inadequate energy availability. It is important to provide precise information to female athletes regarding the effects of inadequate energy on sports performance and health. Therefore, regarding female athletes during puberty, the team coaches and staff can compare the athletes’ body weight to the percent of ideal body weight as a simple screening method for LEA.

The present study has some limitations. First, the sample size was small, and the sports background varied. The sport of gymnastics is associated with a very high impact due to the landings incurred, and gymnasts generally have relatively high BMD even if they have LEA or menstrual irregularities [40]. However, in our study, there were no significant differences in bone parameters between athletes and gymnasts (data not shown). Second, this was a cross-sectional study, and it takes several years to influence BMD according to nutrition and menstrual status [4]. Further longitudinal investigations are required to resolve this problem.

## 5. Conclusions

In the present study, we investigated the relationship between LEA, bone mineral density, and trabecular bone microarchitecture in pubescent female athletes. The LEA was measured using the percent of ideal body weight negatively correlated with bone mineral density and trabecular bone score. These findings indicate that LEA measured using the percent of ideal body weight may be a straightforward and reliable indicator of low bone mineral density and impaired trabecular bone structure among female pubescent athletes. Future prospective studies with larger sample sizes are required to confirm whether LEA can predict low bone mineral density and stress fractures in pubescent female athletes.

## Figures and Tables

**Table 1:** Characteristics of participants (*n* = 21).

Parameter	Mean	SD	Med	Min	Max
Age (years)	14.26	0.73	14.29	12.66	15.56
Height (cm)	157.13	6.01	156.50	147.80	167.90
Weight (kg)	46.65	6.86	44.50	34.00	61.20
Percent of ideal body weight (%)	94.80	8.17	93.30	80.00	110.00
Body fat (%)	19.11	3.53	19.70	14.00	24.90
Fat free mass (kg)	35.50	4.01	35.07	29.09	44.45
Maturity offset (years)	1.42	0.65	1.42	−0.46	2.79
Training year (years)	7.36	2.32	7.00	4.50	11.00
Energy intake (kcal)	2046.28	346.78	2092.00	1470.00	2918.00
Exercise energy expenditure (kcal)	951.74	247.51	917.70	530.25	1593.90
Energy availability (kcal/kg FFM)	31.80	14.96	26.86	10.14	57.89
EAT 26 score	9.67	6.33	8.00	0.00	24.00
Bone mineral content: TBLH (g)	1398.51	191.69	1385.58	1075.75	1736.86
Bone mineral content: TBLH (g/cm^2^)	0.95	0.06	0.95	0.82	1.05
Bone mineral density Z-score: TBLH	0.53	0.75	0.50	−0.90	1.70
Bone mineral content: lumbar spine (g)	42.07	6.66	42.04	31.18	55.11
Bone mineral density: lumbar spine (g/cm^2^)	1.01	0.10	1.00	0.85	1.18
Bone mineral density Z-score: lumbar spine	0.40	0.90	0.50	−1.20	1.90
Trabecular bone score	1.43	0.08	1.41	1.31	1.58

SD: Standard deviation; Med: Median; Min: Minimum; Max: Maximum.

**Table 2:** Characteristics of participants divided into LEA groups.

Parameter	<90% Ideal Body Weight (*n* = 6)		≥90% Ideal Body Weight (*n* = 15)		*p*-Value	<30 kcal/kg FFM (*n* = 12)		≥30 kcal/kg FFM (*n* = 9)		*p*-Value
Age (years)	13.67	(0.52)	13.73	(0.88)	0.87	13.83	(0.94)	13.56	(0.53)	0.44
Height (cm)	153.53	(4.34)	158.57	(6.10)	0.08	159.63	(5.86)	153.80	(4.63)	0.02 *
Weight (kg)	39.93	(3.78)	49.33	(5.93)	<0.01 *	49.67	(6.58)	42.62	(5.14)	0.02 *
Percent of ideal body weight (%)	0.85	(0.03)	0.99	(0.06)	<0.01 *	0.98	(0.08)	0.91	(0.08)	0.06
Body fat (%)	17.77	(2.58)	19.65	(3.78)	0.28	20.43	(3.48)	17.34	(2.89)	0.04 *
FFM (kg)	31.48	(1.94)	37.10	(3.46)	<0.01 *	36.98	(3.97)	33.52	(3.29)	0.05
Maturity offset (yeas)	1.14	(0.23)	1.54	(0.73)	0.22	1.59	(0.79)	1.21	(0.33)	0.19
Energy intake (kcal)	2107.73	(363.04)	2021.70	(349.93)	0.62	1832.93	(226.81)	2330.74	(265.68)	<0.01 *
Exercise energy expenditure (kcal)	791.43	(114.56)	1015.86	(259.64)	0.06	1073.89	(241.28)	788.86	(144.52)	0.01 *
EA (kcal/kg FFM)	42.11	(14.72)	27.67	(13.35)	0.04 *	20.89	(6.27)	46.33	(9.43)	<0.01 *
EAT 26 score	6.00	(3.95)	11.13	(6.60)	0.15	11.17	(7.16)	7.67	(4.66)	0.38
TBLH BMD Z-score	−0.35	(0.37)	0.89	(0.54)	<0.01 *	0.74	(0.70)	0.26	(0.77)	0.15
Lumbar BMD Z-score	−0.60	(0.17)	0.79	(0.75)	<0.01 *	0.55	(0.99)	0.19	(0.79)	0.38
TBS	1.36	(0.03)	1.45	(0.08)	0.01 *	1.44	(0.08)	1.42	(0.08)	0.58

Data are presented as means (SD). FFM, fat-free mass; EA, energy availability; BMD, bone mineral density; TBLH, total body less head; TBS, trabecular bone score. * indicates *p* < 0.05

**Table 3:** Correlation coefficients of bone parameters, physical characteristics, and energy parameters (*n* = 21).

Parameter	TBLH BMD Z-Score	Lumbar BMD Z-Score	TBS
Weight (kg)	0.45 *	0.45 *	0.39
Fat mass (kg)	0.32	0.19	0.24
Fat free mass (kg)	0.47 *	0.57 *	0.45 *
Maturity offset (years)	0.04	0.20	0.27
Energy intake (kcal)	−0.05	−0.09	−0.12
Exercise energy expenditure (kcal)	0.16	0.21	0.09
EAT-26	−0.05	−0.07	0.34

BMD: Bone mineral density; TBLH: Total body less head; TBS: Trabecular bone score. * indicates *p* < 0.05.

## Data Availability

The datasets in this study are available upon reasonable request to the corresponding author’s e-mail.
